# Chemical characterization and gut microbial response unveil modification of polystyrene polymer in the lesser mealworm

**DOI:** 10.1038/s41598-026-44113-3

**Published:** 2026-03-16

**Authors:** Felice Zarra, Rebecca Funari, Claudio Cucini, Cristina Panti, Matteo Baini, Annalaura Brai, Laura Marri, Francesco Nardi, Antonio Carapelli, Francesco Frati

**Affiliations:** 1 Department of Life Sciences, University of Siena, 53100 Siena, Italy; 2 National Biodiversity Future Center (NBFC), 90133 Palermo, Italy; 3 Department of Physical Sciences, Earth and Environment, University of Siena, 53100 Siena, Italy; 4 Department of Biotechnology, Chemistry and Pharmacy, University of Siena, 53100 Siena, Italy

**Keywords:** Plastivorous insects, Plastic degradation, Metabarcoding, Micro-FTIR, GC-MS metabolites, Alphitobius diaperinus, Biological techniques, Biotechnology, Environmental sciences, Microbiology

## Abstract

The massive production of plastics, together with inadequate waste management, has intensified the problem of plastic pollution. Recently, the scientific community has increasingly investigated the interactions between plastic polymers and biological systems, with particular interest in plastivorous insects and their gut-associated microbial communities. The lesser mealworm *Alphitobius diaperinus* has emerged as a promising model for studying biological interactions associated with polystyrene (PS) modification during gut transit. In this study, two complementary approaches were applied to characterize gut mediated responses to PS. First, chemical analyses were employed to provide evidence of polymer modification following gut transit; micro-FTIR analysis confirmed the presence of PS residues in larval frass and showed detectable structural changes in the recovered material, while GC-MS analysis identified α-methylstyrene and cumyl alcohol as compounds associated with PS chemical modifications and not detected in controls. Second, gut microbiota composition was assessed through metabarcoding analysis using full-length PacBio HiFi sequencing of the *16S* rDNA gene. Differences were observed between the microbiota of PS fed larvae and that of the control group under different experimental conditions. Together, these results indicate that PS ingestion leads to measurable chemical modifications of the polymer and shifts in gut microbial composition, providing insight into insect–microbe interactions associated with PS transformation.

**Supplementary Information:**

The online version contains supplementary material available at 10.1038/s41598-026-44113-3.

## Introduction

Plastic pollution has emerged as one of the most critical environmental issues worldwide. Although the negative effects on ecosystems and biodiversity are well known, global plastic production continues to increase, rising from 370.6 million tonnes in 2018 to 430.9 million tonnes in 2024^1,2^. Plastic materials are continuously used given their exceptional properties, such as water resistance, strength, durability, and low cost^3^. The widespread use of plastics across multiple sectors, particularly in packaging industries and for single use products, has led to the generation of an enormous amount of plastic litter over the years^4^. Due to inefficient waste management particles, disposal in landfills or accidental dispersion, large amounts of plastic litter end up in the environment, where they eventually break down into small fragments known as micro-nanoplastics (MNPs), ranging in size from 5 mm to 1 nm. These particles can absorb and transport toxic substances (plastic additives as well as persistent, bioaccumulative and toxic compounds). Once ingested by the organisms and introduced in the food chain, these substances may accumulate in tissues and cause toxicological effects such as inflammation, interfere with metabolism and cellular damage^5–7^.

Due to the environmental risks linked to landfilling, inadequate disposal methods, such as incineration, and the limited efficiency of current recycling methods, the scientific community has increasingly focused on plastic biodegradation processes occurring within biological systems as a potential source of environmentally sustainable solutions. In this context, insects have demonstrated a notable capability to ingest and degrade plastics^8^ with this ability being particularly evident in larval stages of certain Lepidoptera and Coleoptera species^7,9^. These larvae have shown the capacity to survive on plastic diets and to ingest, degrade, and mineralize various plastic materials by passing them through the intestinal tract^9^. The insect gut functions as a natural bioreactor, where physical and biochemical mechanisms contribute to plastic degradation. These include an initial mechanical breakdown (biodeterioration) with subsequent enzymatic depolymerization by gut-associated microorganisms (biofragmentation), which may be followed by microbial assimilation^10–13^.

Among the different types of plastic waste, polystyrene (PS) has received particular attention due to its widespread use and resistance to degradation. Several species in the Tenebrionidae family have shown the ability to ingest and partially degrade PS^9^. One of the earliest studies in this field^14^, demonstrated that the yellow mealworm *Tenebrio molitor* could actively chew and ingest PS. From the gut of larvae fed exclusively on PS, researchers isolated *Exiguobacterium* sp. YT2, a strain capable of adhering to PS surfaces, forming biofilms, and contributing directly to polymer degradation. A later study^15^ showed that the dark mealworm *Tenebrio obscurus* exhibits a substantially higher efficiency in degrading PS. Analysis of its gut microbiota revealed the presence of bacterial taxa belonging to Enterococcaceae, Spiroplasmataceae, and Enterobacteriaceae, which were suggested to play a role in the degradation process. Lately, superworms *Zophobas atratus*^16,17^ were found to consume even larger amounts of PS, compared to other species. This degradative effect was reduced when larvae were treated with antibiotics, suggesting the involvement of the gut microbiota. Further investigation on *Z. atratus* suggested a role for *Pseudomonas aeruginosa* strain DSM 50071, which was found to be capable of colonizing and metabolizing PS surfaces. In the red flour beetle *Tribolium castaneum*, ingestion of extruded PS was also observed^18^. The gut microbiota of these beetles included *Acinetobact*er sp. AnTc-1, a strain with demonstrated PS-degrading potential. Similarly, the darkling beetle *Plesiophthalmus davidis* was shown to host *Serratia* sp. strain WSW, a bacterium capable of forming biofilms on PS surfaces and contributing to its breakdown^19^. These studies collectively demonstrate that several tenebrionid insects can ingest PS and promote polymer degradation under specific experimental conditions.

A further insect model of interest in studies on PS-insect interactions, another member of the Tenebrionidae family, is the lesser mealworm *Alphitobius diaperinus* (Panzer, 1797). Morphologically, *A. diaperinus* presents distinct characteristics across its developmental stages. The larvae have an elongated and segmented body, reaching about 11 mm at full development (Supplementary Figure S1a)^20^. They pass through approximately six to eleven larval instars, depending on the environmental conditions such as temperature and humidity^21^. At the end of the larval development, individuals pupate and complete metamorphosis. Adults emerge from the pupal stage with a broadly oval body, black or brownish, and with a length of approximately 6 mm (Supplementary Figure S1b)^20^. It was recently shown that *A. diaperinus* is able to chew and ingest PS. Metabarcoding analyses revealed significant differences in the gut microbiota of larvae reared on PS foam compared with those maintained on a standard vegetable-based control diet (CT)^22^. A second study examined the gut bacterial community associated with the plastic-eating *A. diaperinus* larvae after an enrichment phase in a medium containing PS as the sole carbon source. After two months, culture-based and molecular analyses revealed the presence of three bacterial strains belonging to the genera: *Klebsiella*, *Pseudomonas*, and *Stenotrophomonas*^23^. Furthermore, the previously *Stenotrophomonas* isolate, which showed the most consistent PS film degradative activity, was fully characterized at the genetic level. Genome sequencing of this isolate, identified as *Stenotrophomonas indicatrix* DAI2m/c, revealed the presence of enzyme encoding genes involved in intracellular metabolic pathways responsible for styrene monomer biodegradation^24^.

Although these studies provided crucial first evidence of PS ingestion and highlighted candidate bacterial taxa, several aspects of the PS degradation process in *A. diaperinus* remain poorly understood. Indeed, to the best of our knowledge, no previous work has examined whether PS ingestion is restricted to specific larval instars, and/or the spatial organization of the gut microbiota along different gut regions. In addition, no study has examined how the polymer changes after gut transit using analytical methods able to detect structural modifications and related by-products. Therefore, this study combines chemical and microbiological approaches to investigate how *A. diaperinus* interacts with PS. The larval instar that ingests the polymer is identified, structural changes to PS after gut transit are assessed through micro-FTIR and GC-MS, and the gut bacterial community is characterised across distinct gut regions using full-length PacBio HiFi *16S* rDNA sequencing. Taken together, these analyses improve the understanding of how developmental stage, gut environment, and microbiota contribute to the partial chemical transformation of PS during gut transit in this species.

## Results

### Identification of the larval instar involved in PS ingestion

In order to evaluate whether all larval instars of *A. diaperinus* are capable of ingesting PS or whether this ability is restricted to specific instars, the cephalic capsule width of larvae collected from PS in five independent rearing replicates was measured (Supplementary Figure S2). Between 47 and 54 larvae entered the PS diet across replicates, and all but three were identified as third Larval Group (LG3) based on cephalic capsule size (Supplementary Figure S3). Within 15 days, 90.3% of these larvae successfully pupated and reached adulthood, confirming their correct identification as LG3 and indicating the general representativeness of the sample. This observation supports the decision to evaluate larval instars separately (see Methods section).

### Assessment of polystyrene modification through chemical analyses

**Fig. 1:**
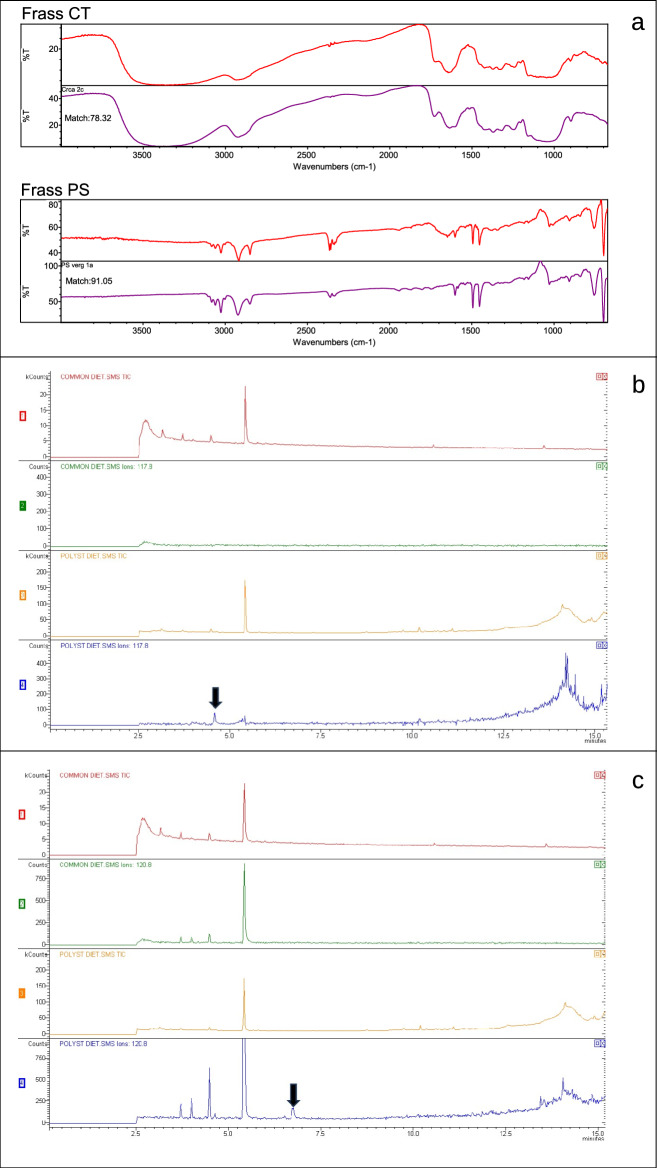
Chemical evidence of polystyrene modification after gut transit in *Alphitobius diaperinus*. (**a**) Micro-FTIR spectra comparing the chemical composition of frass particles with the corresponding feedstock materials. Spectra are shown separately for control diet (Frass CT; upper group) and polystyrene-fed larvae (Frass PS; lower group), as indicated in the figure. Within each condition, upper spectra (red) represent feedstock samples (control diet components or virgin PS) analyzed prior to ingestion, while lower spectra correspond to particles recovered from frass. For each condition, the spectrum showing the closest similarity to the average match score (calculated across replicates and measurements) was selected for visualisation. Match percentages indicate spectral similarity between frass and feedstock materials. (**b**) GC-MS extracted ion chromatograms (EIC) of α-methylstyrene in larvae fed with the control diet (CT) and polystyrene (PS). Panels show the total ion chromatogram (TIC) and the corresponding EIC; arrows indicate the α-methylstyrene peak. (**c**) GC-MS extracted ion chromatograms (EIC) of cumyl alcohol in larvae fed with the control diet and polystyrene . Panels show the TIC and the corresponding EIC; arrows indicate the cumyl alcohol peak.

In order to investigate the chemical composition and assess potential molecular alterations in the PS polymer after gut transit, micro-FTIR spectroscopy was performed on frass samples obtained from LG3 larvae reared on the control diet (LG3-CT) or on PS (LG3-PS). All analyses provided particles of measurable size (50–1,000 μm), producing interpretable spectra suitable for comparison. To confirm the presence of food particles in the frass, and specifically of PS, the spectra of food and frass were compared under different analytical conditions. The similarity analysis showed that bran and carrots had a mean match of 78.37 ± 3.69% with frass from LG3-CT larvae, while virgin PS had a match of 91.61 ± 3.91% with frass from LG3-PS larvae (Fig. 1a). To further validate these results, frass samples were also analyzed after pretreatment with the digestive solutions H₂O₂ and KOH, which reduced excess organic background. In these conditions, the match between virgin PS and frass from LG3-PS larvae was 87.60 ± 7.9% and 91.41 ± 5.46%, respectively (Supplementary Figure S4).

GC-MS analysis was performed to detect potential PS byproducts associated with polymer modification in larvae of *A. diaperinus*. The linearity of the developed method was above 0.99 for all the analytes, and the limit of detection (LOD) ranged from 0.01 mg/kg for styrene to 0.1 mg/kg for cumyl alcohol. The recoveries from the matrix were above 93% for all analytes. Among the five metabolites previously identified as relevant by^25^ and tested here, α-methylstyrene (Fig. 1b) and cumyl alcohol (Fig. 1c) were detected in larvae fed with PS but were absent in those fed with the CT. Both compounds were not detected in PS only controls analyzed under the same extraction and GC–MS conditions (data not shown).

### Characterization of gut microbiota composition in larval instars

**Fig. 2:**
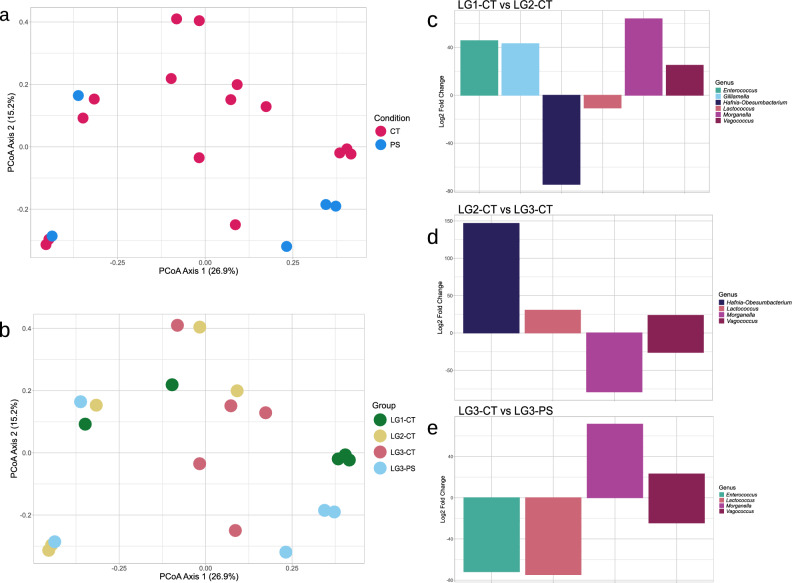
Beta diversity and genus level differential abundance analyses in larval instars. Principal Coordinates Analysis (PCoA) plots visualizing beta diversity among microbial communities: (**a**) comparison between control (CT) and polystyrene fed (PS) groups; (**b**) clustering of microbial communities across individual experimental groups (LG1-CT, LG2-CT, LG3-CT, and LG3-PS). Bar plots showing bacterial genera differentially abundant across larval instars and between feeding conditions: (**c**) differentially abundant genera between LG1-CT and LG2-CT; (**d**) LG2-CT and LG3-CT; (**e**) LG3-CT and LG3-PS. Positive log2 fold change values indicate genera enriched in (**c**) LG2-CT, (**d**) LG3-CT, and (**e**) LG3-PS, while negative values indicate genera enriched in (**c**) LG1-CT, (**d**) LG2-CT, and (**e**) LG3-CT.

To assess whether PS ingestion is associated with changes in specific bacterial taxa across larval development, gut microbiota composition was analyzed across larval instars under control and PS diets. A total of 726,619 high-quality HiFi reads were obtained, showing a per-sample range of 20,207 to 27,445. Alpha diversity indices (Shannon and Simpson) were calculated to evaluate microbial richness and evenness across larval instars (Table 1). No statistically significant differences were observed across instars overall (Shannon: p > 0.05; Simpson: p > 0.05), suggesting a relatively stable microbial diversity. Beta diversity was assessed at two distinct levels of comparison and visualized through PCoA plots. The first level focused on differences in microbial communities between CT and PS groups (Fig. 2a), while the second examined the clustering across all experimental groups (LG1-CT, LG2-CT, LG3-CT, and LG3-PS) (Fig. 2b). PERMANOVA (Permutational Multivariate Analysis of Variance) results for both comparisons indicated that the observed differences were not statistically significant (p.adj > 0.05). The associated R^2^ values were low to moderate (global CT vs PS: R^2^ = 0.054; pairwise R^2^ = 0.086–0.183; Supplementary Table 2). All PERMANOVA p-values correspond to Benjamini–Hochberg adjusted values. While overall community structure did not differ significantly between diets across larval instars, analyses at the ASV level revealed diet-associated changes in specific bacterial taxa. For a more comprehensive overview of the microbial community composition across larval instars, additional details are provided in the Supplementary Material (Supplementary Figures S5–8). The Venn diagram, which provides a descriptive overview of ASVs across different conditions, showed that 34 ASVs constituted the core shared among all groups, while other ASVs were identified only within individual instars (17 in LG1-CT, 13 in LG2-CT, 34 in LG3-CT, and 37 in LG3-PS). Only one ASV was shared exclusively between LG3-CT and LG3-PS (Supplementary Figures S5). These presence–absence patterns are descriptive and were not used to infer differential abundance, which was assessed using DESeq2 (see Methods section). Phylum-level community composition across larval instars under control and polystyrene diets is reported in the.Supplementary Figure S6. The taxa bar plot and heatmap revealed a relatively homogeneous microbial community across larval instars. *Enterococcus* and *Corralincola* were abundant in the different replicates but almost exclusively associated with the control groups, whereas *Lactococcus*, *Enterobacter*, *Hafnia-Obesumbacterium*, and *Vagococcus* appeared more consistently across all groups, representing a stable core microbiota (Supplementary Figures S5 and 8). Differential abundance analysis confirmed these patterns, showing *Enterococcus* and *Lactococcus* as significantly more abundant in control samples. In the crucial comparison of whole LG3 guts between CT and PS diets, *Morganella* was detected exclusively in the PS group and absent in the CT group (Fig. 2c–e). Differential abundance analysis was performed at the ASV level; for visualization purposes, bar plots summarize genus-level trends, while the full list of significantly enriched ASVs, including log₂ fold change and adjusted p-values, is reported in Supplementary Table 3.

**Table 1:** Alpha diversity indices (Shannon and Simpson) calculated across larval instars and gut sections under control (CT) and polystyrene (PS) conditions. Values are reported as mean ± standard deviation.

**Metabarcoding analysis**	**Diet group**	**Sample (larval group / gut section)**	**Shannon diversity index**	**Simpson diversity index**
Larval instars	CT	LG1	2.53 ± 0.69	0.83 ± 0.10
Larval instars	CT	LG2	2.51 ± 0.21	0.88 ± 0.04
Larval instars	CT	LG3	2.67 ± 0.69	0.85 ± 0.13
Larval instars	PS	LG3	2.59 ± 0.21	0.90 ± 0.02
Gut sections	CT	Foregut	2.99 ± 0.61	0.92 ± 0.08
Gut sections	CT	Midgut	2.48 ± 0.55	0.84 ± 0.05
Gut sections	CT	Hindgut	2.85 ± 0.34	0.90 ± 0.03
Gut sections	PS	Foregut	1.58 ± 0.02	0.66 ± 0.00
Gut sections	PS	Midgut	2.30 ± 0.83	0.78 ± 0.10
Gut sections	PS	Hindgut	3.11 ± 0.11	0.94 ± 0.01

### Characterization of gut microbiota composition in gut sections

**Fig. 3:**
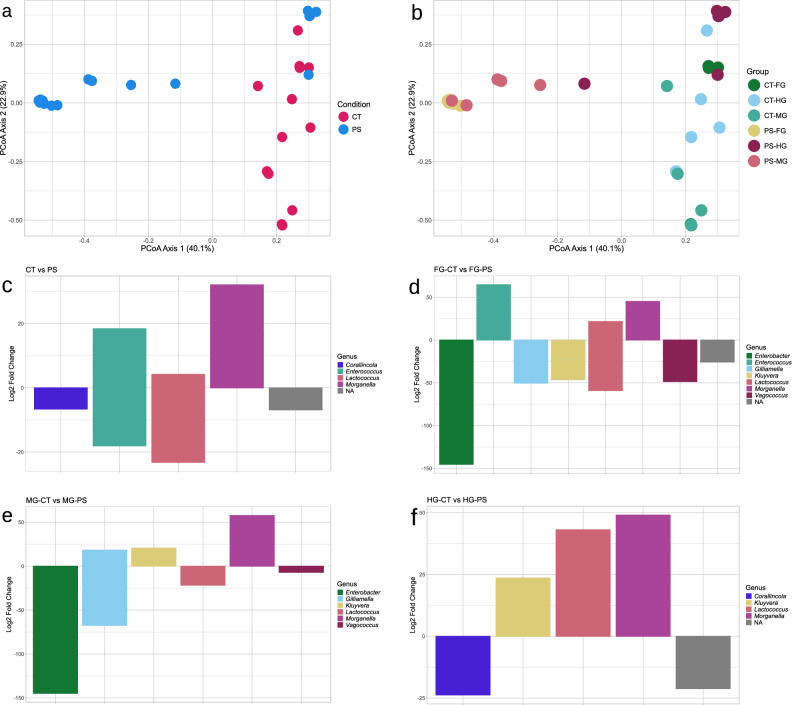
Beta diversity and genus level differential abundance analyses in gut sections. Principal Coordinates Analysis (PCoA) plots visualizing beta diversity among microbial communities: (**a**) comparison between control (CT) and polystyrene fed (PS) groups; (**b**) clustering of microbial communities across individual gut sections (FG-CT, MG-CT, HG-CT, FG-PS, MG-PS, and HG-PS). Bar plots showing bacterial genera differentially abundant between diets and within gut sections: (**c**) entire gut (CT vs PS); (**d**) foregut (FG-CT vs FG-PS); (**e**) midgut (MG-CT vs MG PS); (**f**) hindgut (HG-CT vs HG-PS). Positive log_2_ fold changes values indicate genera enriched in PS fed groups, while negative values indicate genera enriched in CT fed groups.

To investigate whether PS feeding is associated with changes in specific bacterial taxa across the gut, microbial community composition was analyzed at both diversity and taxonomic levels in distinct gut sections under control and PS diets. A total of 471,734 high-quality HiFi reads were obtained, with a per-sample range of 20,434 to 27,378. Alpha diversity analysis revealed significant differences among gut sections and feeding treatments (both Shannon: p < 0.05; Simpson: p < 0.05), indicating that microbial community complexity varied according to both anatomical region and diet (Table 1). Beta diversity analysis was conducted at two levels of comparison. The first comparison, between CT and PS groups (Fig. 3a), showed a statistically significant separation (p.adj < 0.05), with R^2^ = 0.235 for this comparison. The second comparison, across all gut sections (FG_CT, MG_CT, HG_CT, FG_PS, MG_PS, HG_PS; Fig. 3b), revealed significant differences between FG_CT and FG_PS (p.adj < 0.05; R^2^ = 0.664) and between MG_CT and MG_PS (p.adj < 0.05; R^2^ = 0.546) but not between HG_CT and HG_PS (p.adj > 0.05; R^2^ = 0.195). R^2^ values for all remaining pairwise contrasts, together with the complete set of PERMANOVA results, are reported in Supplementary Table S2. No significant differences were detected across gut sections within CT. In contrast, within the PS group, significant differences were observed between FG_PS and HG_PS (p.adj < 0.05) and between MG_PS and HG_PS (p.adj < 0.05). A complete summary of PERMANOVA results is provided in Supplementary Table 2. Supplementary figures offer further details on the microbial community composition across gut sections (Supplementary Figures S9–12). The Venn diagrams provided a descriptive overview of ASVs distribution between CT and PS groups. In the CT group, the highest number of ASVs (47) was shared among the three gut sections, while unique ASVs were identified in FG (22), MG (21), and HG (42). In contrast, the PS group showed a markedly higher total number of ASVs, with only 25 of them shared across all gut sections. Unique ASVs were more abundant, with 17 detected in FG, 339 in the MG, and 64 in the HG (Supplementary Figure S9). These descriptive patterns were not used for differential abundance inference, which was in turn assessed using DESeq2 (see Methods section). Phylum-level community composition across gut sections under control and polystyrene diets is reported in the Supplementary Figure S10, providing a higher-level overview of taxonomic shifts associated with PS feeding. The taxa bar plot and heatmap indicated that *Hafnia-Obesumbacterium*, *Enterobacter*, and *Kluyvera* were consistently present across gut sections and diets. In contrast, *Vagococcus*, *Enterococcus*, and *Corralincola* predominated in the control groups, whereas *Morganella* was particularly enriched in PS-fed larvae (Supplementary Figures S11 and 12). Differential abundance analysis of whole LG3 larvae, without separating gut sections, confirmed the presence of *Enterococcus* and *Lactococcus* in both diets, with *Corralincola* restricted to CT and *Morganella* exclusively detected in PS (Fig. 3c). When comparing gut sections within the PS group, specific genera were identified: *Enterococcus* and *Morganella* in the foregut, *Kluyvera* and *Morganella* in the midgut, and *Kluyvera*, *Morganella*, and *Lactococcus* in the hindgut (Fig. 3d–f). Quantitative support for these patterns is provided by the ASV-level DESeq2 results reported in Supplementary Table 3.

## Discussion

The widespread use of plastics and inadequate waste management strategies have contributed to plastic pollution in the environment. A promising mitigation to the problem could be biodegradation, which involves the breakdown of plastics through the combined action of plastivorous insects and their associated gut microbiota. In this context, *A. diaperinus* has attracted interest as an experimental model to study PS ingestion and associated biological responses, as reported in a previous study. Cucini et al.^22^ demonstrated that this species can chew and ingest PS, while showing important differences in gut microbiota composition between larvae fed with PS and those fed with a vegetable-based diet. The same authors also reported high mortality rates and reduced larval weight under PS diets, findings later confirmed by a further study on the same species^26^.

Initially, it was assessed whether all larval instars of *A. diaperinus* are capable of ingesting PS and surviving under such feeding conditions, or whether this ability is restricted to specific moments of larval development. The results demonstrated that PS ingestion occurs exclusively in the last larval instar (LG3, corresponding to VII-VIII larval instars, see Supplementary Table 1), as confirmed by cephalic capsule measurements. This developmental instar appears predisposed to polymer ingestion, probably because the material is perceived as a site for pupation and adult transition. In the light of the difficulties reported by Cucini et al.^22^ and Richard et al.^26^ regarding the reduced viability of *A. diaperinus* colonies reared exclusively on PS, this observation may have practical implications. It suggests that colonies could be maintained on a standard diet to ensure full viability and then transferred to a PS diet only at the LG3 stage, thus maximizing their exposure to PS during the developmental stage in which ingestion occurs. This finding is also relevant for metabarcoding analyses, because it directs attention to the developmental instar effectively involved in degradation, as shown by the experimental setup used in this study.

Chemical analyses were applied to evaluate structural modifications of the polymer after gut passage. Micro-FTIR analysis confirmed the ingestion of PS by detecting polymer residues in the frass and showed that the polymeric structure retained about 90% similarity to the original material. GC-MS analysis yielded additional evidence, detecting α-methylstyrene and cumyl alcohol in PS fed lyophilized larvae, both absent in controls. The detection of α-methylstyrene, a compound often associated with the depolymerization of PS, provides evidence of chemical modifications occurring during gut transit. This observation aligns with findings in *T. molitor* reported by Tsochatzis et al.^25^, where the formation of α-methylstyrene was attributed to enzymatic or microbial activity within the gut in that experimental system. The detection of cumyl alcohol, another key metabolite, suggests a gradual degradation process where oxidative or enzymatic reactions contribute to PS transformation. Comparable evidence has been reported in *A. diaperinus* by Richard et al.^26^, who detected oxidation products in PS frass from both larvae and adults using FTIR spectroscopy and Py-GC/MS. To exclude the abiotic origin of the two metabolites, the PS material itself was analyzed using the extraction method by Garrigós et al.^27^. The absence of α-methylstyrene and cumyl alcohol in PS-only controls, even at the highest analyzed concentration (14 mg/mL, in line with larval ingestion data reported in Cucini et al.^22^) indicates that these metabolites are not formed spontaneously during extraction. Taken together, these findings indicate that the α-methylstyrene and cumyl alcohol detected in larval extracts are of biological origin and are associated with gut passage rather than with the polymer material alone. Their occurrence during digestion suggests that chemical modification of PS takes place within the gut environment. Although direct functional attribution cannot be made on the basis of the present data, the detection of these metabolites in PS-fed larvae supports the involvement of gut-associated biological processes, potentially including microbial activity, in PS transformation. This evidence indicates that exposure to the polymer alone is insufficient to explain the presence of α-methylstyrene and cumyl alcohol, pointing instead to the role of the larval gut system in their formation.

The present study supports these observations by confirming the presence of known depolymerization related metabolites and by quantifying a measurable, although partial, alteration in the polymer structure. These results indicate that PS undergoes detectable chemical modifications during gut transit, while largely retaining its original structure. Taken together, micro-FTIR and GC–MS analyses show a high degree of spectral similarity, suggesting only partial alteration of the polymer backbone. However, GC–MS analysis revealed the presence of two low-molecular-weight compounds commonly associated with polystyrene degradation, which were detected exclusively in PS-fed larvae. This provides clear evidence that polystyrene undergoes limited but detectable chemical modification during gut transit. Such modifications occur within the complex biological environment of the larval gut, where digestive and biological processes act simultaneously on the polymer. On this basis, a metabarcoding approach was used to investigate whether PS ingestion is associated with changes in gut microbial community composition across larval instars and along different gut regions under the different experimental conditions considered in this study. This analysis focused on diet-associated patterns in gut microbiota composition. Comparisons across larval instars did not reveal significant differences in microbial diversity across larval groups, indicating that the core microbiota remains relatively stable throughout development and across diets. Accordingly, PS feeding does not appear to induce the overall gut community structure across developmental instars (Fig. 2a–b), although it may influence the relative abundance of specific taxa (Fig. 2c–e; Supplementary Table 3). In contrast, the analysis of gut sections revealed marked differences. In controls, microbial diversity was uniform across foregut, midgut, and hindgut. In PS fed larvae, however, diversity increased progressively along the tract, with the highest values in the hindgut. Beta diversity confirmed these differences, revealing distinct separation between the two dietary groups, especially in the foregut and midgut. Within the PS fed group, the three gut sections were also different in their microbial profiles, with the most marked difference observed between fore-/mid- and hindgut. These results suggest that the impact of PS differs across gut sections, producing local changes in microbial communities. While diversity metrics provided a general overview of community structure, changes in gut microbiota were further examined at the genus level, focusing on bacterial taxa consistently enriched under PS-feeding conditions. Differential abundance analysis provided further insights about the microbial community composition across larval instars and gut sections. A stable core community was identified across all groups, including genera such as *Lactococcus*, *Vagococcus*, *Enterococcus*, and *Gilliamella*, that were consistently detected in both diets. The relevance of specific taxa became evident mainly in comparisons involving PS fed groups, which represent the crucial context for PS exposure and transformation. Some genera, such as *Hafnia-Obesumbacterium*, *Enterobacter*, and *Corallincola*, were abundant only in CT groups. In PS fed larvae, *Morganella* emerged as the most represented genus, being consistently detected at higher levels both across larval instars and in gut sections. *Kluyvera* was also enriched, especially in the midgut and hindgut. The recurrence of these genera under PS feeding conditions indicates that they are consistently associated with the microbial response to polymer ingestion. Although the present data do not allow a direct attribution of a functional role in polymer transformation to *Morganella* or *Kluyvera*, both taxa have been identified in polystyrene degradation in other plastivorous insects’ guts. In particular, *Morganella morganii* has been reported as a major component of the gut microbiota of *Z. atratus* fed on expanded polystyrene, with its abundance increasing under supplementation with microelements and vitamins^28^. In *T. molitor*, its prevalence also increased when the larvae were fed polystyrene subjected to specific aging treatments, such as UV exposure combined with freezing^29^. Similarly, *Kluyvera* has been associated with plastic degradation in *Z. atratus*, where it was more abundant in larvae fed on expanded or extruded polystyrene compared to cereal fed controls^30^. Ndotono et al.^31^ reported similar findings, identifying *Kluyvera* under PS-enriched diet conditions, which supports its consistent association with PS exposure. They additionally found *Enterococcus* and *Lactococcus* to be prevalent in the PS group, while in the current study, these genera were present in both diets. No bacterial taxa identified in this study correspond to those reported by Cucini et al.^22^. This discrepancy can be attributed to variations in experimental conditions, including larval instar analyzed, gut sections considered, rearing conditions, and sequencing techniques employed, all of which are known to influence gut microbiota composition in insects.

In a previous study we isolated a *Stenotrophomonas* sp. strain from PS-fed larvae of *A. diaperinus* after two-months enrichment on carbon free medium with PS as sole carbon source^23^. The strain was subsequently characterized as a potential degrader of PS and is currently available at the CRBIP (France) and DSMZ (Germany) culture collections under the accession number CIP 112583 and DSM 120327, respectively^24^. In the present investigation, *Stenotrophomonas* taxon was detected only sporadically in one LG3-CT library, and additional searches on raw reads confirmed its presence at very low abundance also in LG3-PS libraries. This finding is unsurprising considering the intense artificial selection in the PS-enriched culture, which served as a strong selective filter favouring bacteria capable to survive and thrive under the highly restrictive conditions over the two-months period. During this time, the community was not only filtered but also became ecologically and potentially genetically distinct from the original inoculum^23^.

The results reported here, derived from the analysis of various groups of PS-fed larvae across different larval instars and gut sections, indicate that the bacterial genera *Morganella* and *Kluyvera* represent stable components of the gut microbiome under PS-feeding conditions, in line with findings from other plastivorous insects^28–30^, without implying a direct involvement in polymer transformation. This suggests that both may be considered as potentially auxiliary taxa involved in the gut microbiome’s response to PS ingestion. The chemical and microbiological results provide a more coherent view of the interaction between PS and the larval gut environment. The detection of PS derived by-products after gut transit, along with the consistent enrichment of specific bacterial taxa in PS-fed larvae, indicates that both polymer modification and shifts in gut microbial composition occur during digestion. Chemical and microbiological analyses address different but complementary aspects of PS processing during gut transit. Chemical data document polymer modification after digestion, whereas microbiota profiles describe changes in the gut microbial community associated with PS exposure. Nevertheless, this evidence remains correlative, and the data do not allow attribution of a direct functional role in polymer transformation to individual bacterial taxa based on *16S* rDNA gene sequencing alone. Consequently, the potential involvement of specific bacterial taxa in PS transformation remains to be validated and will require the isolation and characterization of candidate taxa, followed by *in vitro* assays, genomic analyses, and functional studies on the enzymes potentially involved in metabolic pathways of polymer breakdown.

This study addressed three complementary aims to investigate the interaction between *A. diaperinus* larvae and expanded polystyrene. We showed that PS ingestion is restricted to the LG3 instar, identifying a defined developmental stage in which larvae are able to ingest and tolerate the polymer. Chemical analyses demonstrated that PS undergoes partial physicochemical modification during gut transit, as indicated by the presence of polymer residues in the frass and by the detection of α-methylstyrene and cumyl alcohol in PS-fed larvae, while largely retaining its original structure. In parallel, metabarcoding analyses revealed that PS ingestion is associated with diet-related changes in gut microbial composition. Although a stable core microbiota was maintained across instars and diets, specific bacterial taxa were identified as significanlty enriched under PS-feeding conditions, particularly in the foregut and midgut of LG3 larvae, indicating a microbial response to PS exposure without implying direct functional roles.

Overall, the findings highlight key chemical and microbiological processes associated with polystyrene transformation during gut transit in plastivorous insects. *Alphitobius diaperinus* was shown to ingest expanded PS, induce partial chemical modification of the polymer , and exhibit associated changes in gut microbial community structure. Taken together, these results indicate that *A. diaperinus* is a useful experimental model for studying plastic-gut interactions under controlled conditions. Any potential application of these findings will require substantial additional work, including functional validation and quantitative assessment of transformation processes, before scalability and practical relevance can be considered.

## Methods

The larvae of *A. diaperinus* used in this study were sourced from Agripet Garden (Padova, Italy; reared according to the factory protocol, see: https://www.agripetgarden.it/). Approximately 5,000 larvae were reared under controlled environmental conditions (20 ± 2 °C; 12-hour light/dark cycle; 50–70% RH) at the Department of Life Sciences of the University of Siena. The expanded polystyrene foam, commercially known as Extir® (CAS 9003-53-6), with a density of 0.01 g/cm^3^, was purchased from Toscoespansi s.r.l. (http://www.toscoespansi.it/). According to the supplier, the material did not contain additional additives or catalysts.

### Identification of the larval instar involved in PS ingestion

An experimental rearing was established in five replicates, each containing 240 larvae, distributed as 80 individuals per Larval Group (LG1, LG2, LG3). The developmental instar of each larva was determined by measuring the cephalic capsule under a stereomicroscope, adapting the classification of Francisco & Do Prado^21^ for more practical purposes (see Supplementary Table 1) by merging multiple larval instars (L) in three larval groups (LG) only. The photographs were taken using a Nikon® D850 digital camera equipped with an AF-S Micro Nikkor 105 mm f/2.8G ED lens and a Zeiss® Axio Zoom.V16 stereomicroscope. The larvae in each rearing setup were provided with a mixed diet consisting of carrots, bran, and a piece of PS. Every three days, PS pieces were inspected for the presence of larvae and those found inside the plastic chunks were measured for cephalic capsule width. Fresh PS pieces were then introduced to continue the procedure. After measurement, larvae were transferred to secondary rearing setups without PS to monitor their progress and observe how many completed their life cycle by reaching adult stage. The procedure was repeated five times.

### Assessment of polystyrene degradation through chemical analyses

Chemical analyses were conducted to compare food before and after ingestion and passage through the insect’s intestinal tract, in order to detect digestion-related chemical alterations. Only LG3 larvae were used, collected directly from the section of PS and CT rearing systems. The rearing was maintained for one month, with emerging adults regularly removed and replaced by new LG3 larvae.

### Micro-fourier transform infrared spectroscopy (micro-FTIR) analysis

The larvae were cleaned of residual PS/CT powder with compressed air and placed in two clean glass boxes overnight to allow frass accumulation. The following day, frass was carefully collected and stored at -20°C. This procedure was repeated for approximately three weeks, as in Yang et al.^32^. Six sample types were processed: PS food and PS frass, each analyzed in native form and after an overnight digestion at room temperature in 15% hydrogen peroxide (H₂O₂, 1:10 w/v), and after digestion in 10% potassium hydroxide (KOH, 1:10 w/v) (modified from [33]). These treatments were applied to reduce excess organic matter and improve spectral acquisition, without affecting the integrity of the polymer. For the main comparative analyses, spectra from native materials and corresponding frass were used, while spectra from pretreated samples are provided as methodological validation (see Supplementary Materials). For each sample, five particles were randomly selected, and three spectra were acquired from each particle (n = 15 spectra per condition). Visual verification and chemical identification of microplastics was carried out with a micro-FTIR (Nicolet iN10 MX Infrared Imaging Microscope, Thermo Scientific) in transmission mode. Micro-FTIR spectra of all particles were recorded in the spectral range from 4000 to 400 cm^−1^ with a collection time of 45s and 128 co-scans for each measurement. The spectral resolution was 4 cm^−1^, and the aperture size was a range of 10×10 μm to 150×150 μm depending on the size of particles. Additionally, *ad-hoc* libraries (based on native and digested material spectra acquisition) and built-in instrument libraries (HR Polymer Additives and Plasticizers, Wizard Library, and Thermo Electron) were used for further verification and analysis of the obtained spectra, using the OMNIC Picta Software (Thermo Scientific). The determination of the polymer was accepted when the spectra of the isolated particle showed a similarity to the library spectra and a Hit Quality Index (HQI) higher than 70 %. For each sample, five particles were analysed and three spectra were collected per particle; all similarity values were extracted from OMNIC Picta and subsequently averaged. For figure visualization, the spectrum whose similarity value was closest to the group mean was selected as representative.

### Gas chromatography–mass spectrometry (GC–MS)

Larvae were collected over a period of approximately two weeks and subsequently lyophilized^25^. A 200 mg portion of the lyophilized larvae was transferred into a 2 mL vial, to which 500 µL of chloroform were added. The sample was homogenized with an IKA Labortechnik T25 basic (IKA Werke GmbH & Co., Staufen, Germany). The sample was vortexed and sonicated at 25°C for 30 minutes in an ultrasound bath (Bandelin Sonorex, Rangendingen, Germany) operating at a frequency of 35 kHz. After centrifugation at 2500 rpm for 5 min, supernatant and pellet were separated and the solvent was recovered. Finally, samples were filtered with PTFE 0.45 mm filters. Chromatographic analyses were performed with a Varian 3900 gas chromatograph with a CP-8400 auto-sampler coupled to Varian Saturn 2000 MS/MS ion trap mass spectrometer (Varian, Palo Alto, CA, USA). The samples were analyzed with a capillary column VF-5ms with the following dimensions: 30 m x 0.25 mm ID, 0.25 µm d.f. (FactorFour, Varian Inc., USA). The analysis was performed using a split/split-less injector at 250 °C in splitless mode, with a column flow of 1.5 mL min^−1^. The injection volume was 1 μl. The oven program was the following: initial temperature of 65 °C for 2 min, ramp (7 °C min^−1^) up to 202 °C, ramp (100 °C min^−1^) up to 275 °C (isothermal of 1 min). The total run time was 23 min, including a solvent delay time of 2.5 min, applying Electron Impact Ionization at 70 eV. Mass analysis and detection were performed using a scan range from 40 to 400 amu. The identification of PS degradation by-products was carried out using the NIST5 library and commercial standards including Styrene ≥98%, α-Methylstyrene ≥98.5%, Acetophenone ≥99.5%, Cumyl alcohol 97%, and 2,4-di-tert-butylphenol ≥99%. All standards and chloroform were purchased from Sigma-Aldrich S.r.l. (Milan, Italy). To verify whether PS by-products were already present in the polymer material, the method developed by Garrigós et al. with minor modifications, was applied^27^. In detail, 7 mg of expanded PS were transferred into a 2 mL vial, mixed with 500 µL dichloromethane, and sonicated at 25°C for 30 min. Then, 250 µL of methanol were added to precipitate the polymer, and the mixture was centrifuged at 2500 rpm for 5 min. The dichloromethane phase was recovered, filtered through 0.45 µm PTFE filters and analyzed by GC using the same method developed for larval tissues.

### Characterization of gut microbiota composition

Metabarcoding analyses were performed to investigate the gut microbiota composition of *A. diaperinus* under different experimental conditions, with particular focus on gut segments and the development instar involved in the process. Larvae were therefore divided into four rearing groups: LG1-CT, LG2-CT, and LG3-CT, consisting of instars LG1, LG2, and LG3 reared on a diet of carrots and bran; and LG3-PS, consisting of LG3 larvae fed exclusively with PS foam.

### Sample preparation

Larvae were externally sterilized by immersion in 75% ethanol for approximately 2 min, rinsed five times with sterile water, and dissected to extract the entire gut. Fatty tissue and Malpighian tubules were removed^34^. For metabarcoding analyses, two sample sets were prepared. Whole guts were used to obtain a general overview of the gut microbiota across larval instars, while dissected guts (foregut, midgut, hindgut) were analyzed to characterize individual gut regions. For each experimental group, five replicates were generated. Each replicate consisted of a pool of three guts or three gut sections, resulting in a total of 20 and 30 samples for the two analyses, respectively. Each replicate derived from a different larva within the same rearing group, and no larva contributed more than one sample to the same type of pool. The pooled sample was treated as the biological unit for all downstream analyses.

### Extraction and sequencing of bacterial DNA

Bacterial DNA was extracted using the QIAamp® PowerFecal® Pro DNA Kit, following the manufacturer’s protocol. The final DNA concentration was measured using both a NanoDrop ND1000V spectrophotometer (NanoDrop Technologies, Wilmington, DE, US) and a Qubit® 4.0 fluorometer (Life Technologies Corporation, Carlsbad, CA, US). The quality of the DNA was assessed by 1% agarose gel electrophoresis to ensure its suitability for downstream analyses. The extracted DNA samples were sent to Biomarker Technologies (BMK) GmbH (Münster, Germany) for sequencing. The samples underwent full-length *16S* rDNA gene amplicon sequencing using the PacBio Revio system in CCS mode (HiFi reads) to obtain high-accuracy long-read sequences, improving the consistency of taxonomic assignment and reducing sequencing errors in comparative analyses across larval instars, gut sections, and dietary conditions. For the amplification of the full-length *16S* rDNA gene, the universal primers 27F (5’-AGRGTTTGATYNTGGCTCAG-3’); 1492R (5’- TASGGHTACCTTGTTASGACTT-3’) were used. The sequencing was performed with a target of 20,000 reads per sample.

### Data analysis

Raw reads were entirely analyzed in the R environment^35^. To infer amplicon sequence variants (ASVs) and remove sequencing errors, the raw reads were processed using the DADA2 package^36^. Primer sequences were removed, and reads were trimmed and filtered by length (1200-1450 bp), quality (maxEE = 10) or ambiguous bases (maxN = 0). Dereplication and error modeling were performed to accurately identify true biological sequences and distinguish them from sequencing errors. Chimeric sequences were identified and removed. Taxonomy assignment was conducted using the SILVA v138.1 database^37^. Given the use of full-length *16S* rDNA gene sequencing with PacBio HiFi technology, high resolution ASVs were inferred without applying clustering thresholds. The relative frequencies of genera (genus frequency) were calculated to determine the distribution and proportion of each genus within the microbial communities. The visualization of genus distribution across the samples was created as bar plots using the ggplot2 package^38^. For downstream analyses, the ASV tables, taxonomy tables, and sample metadata were integrated into a phyloseq object^39^. Alpha diversity was evaluated using Shannon and Simpson indices. To assess whether alpha diversity significantly differed between experimental groups, a Kruskal-Wallis test was performed separately for each index. A p-value threshold of 0.05 was used to determine statistical significance. Beta diversity was evaluated using the Bray-Curtis distance metric. The separation of microbial communities across experimental groups was visualized using Principal Coordinates Analysis (PCoA), with visual outputs produced in ggplot2. Statistical differences in community composition were tested using PERMANOVA via the pairwiseAdonis package^40^. Significance was determined using 999 permutations, and p-values were adjusted for multiple testing using the Benjamini-Hochberg correction. Differential abundance analysis was conducted using DESeq2 package^41^ to identify taxa significantly enriched or depleted between experimental groups. Results were filtered to include only taxa with p.adj < 0.05. The presence and distribution of ASVs across experimental groups were examined using Venn diagrams generated with the VennDiagram package^42^. The taxonomy of shared and unique ASVs among the groups examined was extracted and saved in CSV files for further interpretation. Plots were generated with ggplot2 package^38^.

## Supplementary Information

Supplementary Information 1.

Supplementary Information 2.

## Data Availability

Raw data were deposited in NCBI’s SRA database within BioProject ID PRJNA1330245, SRA numbers: SRS26581475, SRS26581478, SRS26581479, SRS26581482, SRS26581483, SRS26581486, SRS26581487, SRS26581489 - SRS26581491, SRS26581495, SRS26581497, SRS26581498, SRS26581502, SRS26581503, SRS26581506, SRS26581509, SRS26581510, SRS26581512, SRS26581515, SRS26581517 - SRS26581520, SRS26581522, SRS26581526, SRS26581528, SRS26581530, SRS26581531, SRS26581535, SRS26581536, SRS26581538, SRS26581539, SRS26581541, SRS26581544, SRS26581548, SRS26581549, SRS26581551, SRS26581552, SRS26581554, SRS26581555, SRS26581558, SRS26581560, SRS26581562 - SRS26581564, SRS26581566, SRS26581571 - SRS26581573, and Bio-Sample numbers: SAMN51481962 - SAMN51482011. All supporting data, code and protocols have been provided within the article, through supplementary data files or on the following link: https://github.com/ESZlab/Alphitobius_metabarcoding.
