# Supplementary material for: Chemical characterization and gut microbial response unveil modification of polystyrene polymer in the lesser mealworm
Source: Sci Rep. 2026 Mar 16;16:13607. doi: 10.1038/s41598-026-44113-3 (PMC13121710; doi:10.1038/s41598-026-44113-3)
Supplement: Supplementary file 1 — Supplementary Information 1. [file 41598_2026_44113_MOESM1_ESM.pdf]

SUPPLEMENTARY FIGURES

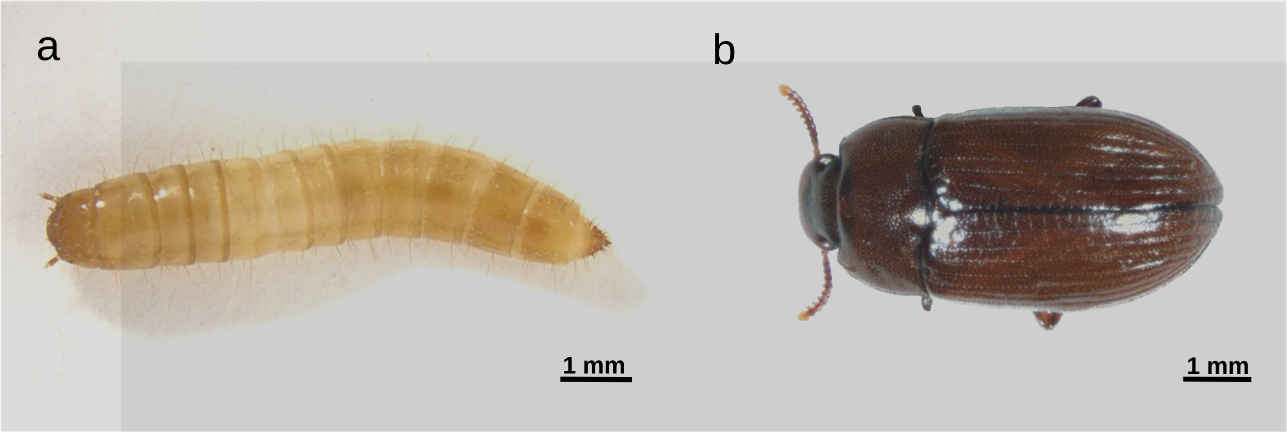

Supplementary Figure S1 - *Alphitobius diaperinus* at different developmental stages. (a) Larva with an elongated, segmented body. (b) Dorsal view of an adult.

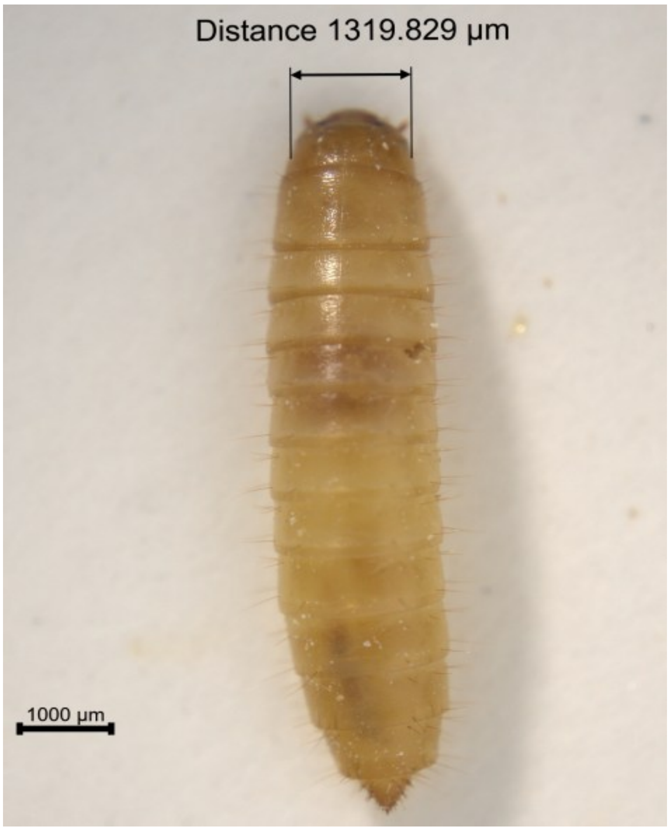

Supplementary Figure S2 - Cephalic capsule of last instar larvae.

Cephalic capsule measurement of last instar (LG3) *Alphitobius diaperinus* larvae performed under a stereomicroscope. The measured width (1319.8 µm) corresponds to the threshold value reported for the final larval instar. Scale bar: 1000 µm.

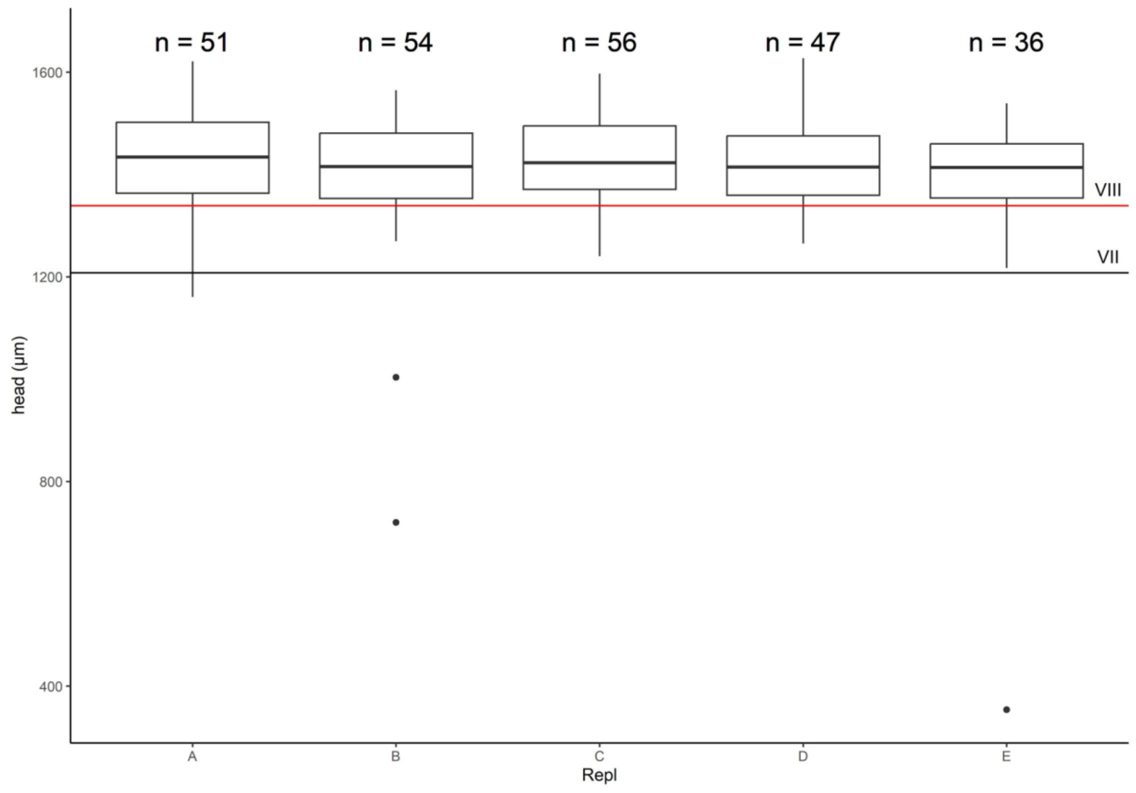

Supplementary Figure S3 - Cephalic capsule width of larvae extracted from polystyrene. Boxplot showing the distribution of cephalic capsule widths for larvae recovered from PS pieces across five rearing replicates (A–E). Horizontal lines indicate thresholds for larval instars according to Francisco & Do Prado [21] (see Supplementary Table S1). The red line corresponds to the value associated with the final (VIII) instar. Numbers above each box represent the total larvae measured in each replicate.

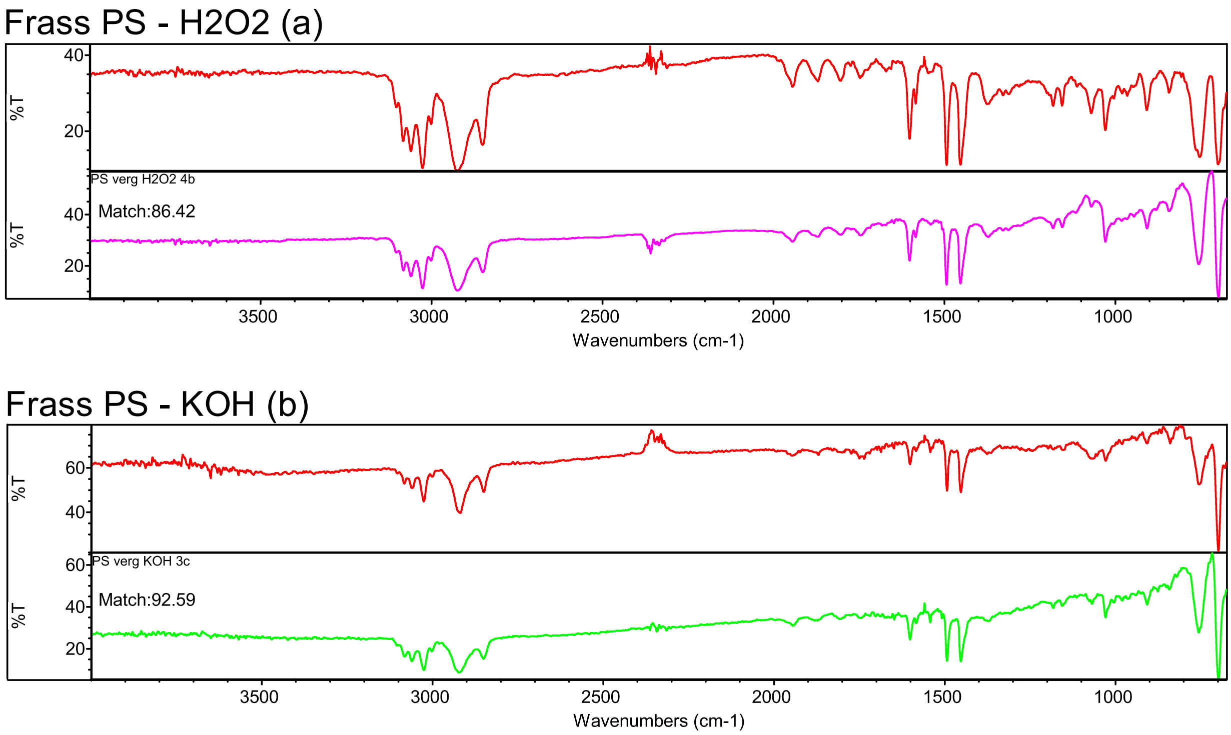

Supplementary Figure S4 – Validation of Micro-FTIR analyses after frass pretreatments. Micro-FTIR spectra of frass from LG3-PS larvae following digestion with H_2O_2 (a) or KOH (b). Pretreatments were applied to reduce excess organic matter and improve spectral acquisition. Virgin PS spectra are shown in red, while frass spectra are shown below. For each condition, the spectrum with the closest similarity to the average match score (calculated across all replicates and measurements) was selected for visualization. The match percentages indicate the similarity between frass and virgin PS spectra

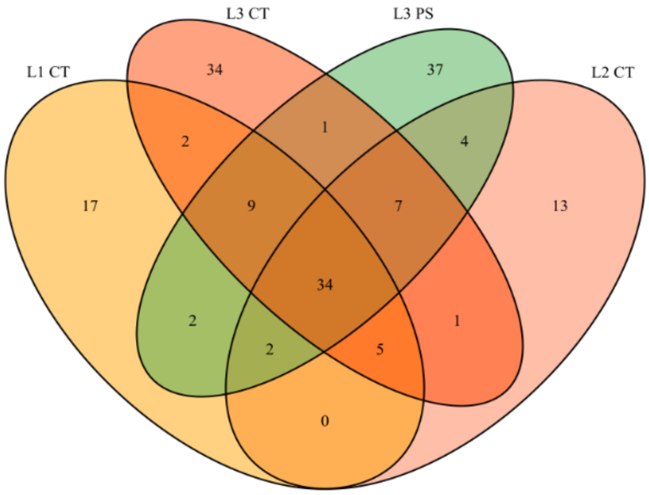

Supplementary Figure S5 – ASV distribution across larval instars.

Venn diagram showing the distribution of ASVs among *A. diaperinus* larvae at different instars (LG1_CT, LG2_CT, LG3_CT, LG3_PS). Numbers represent ASVs shared between groups or unique to each condition.

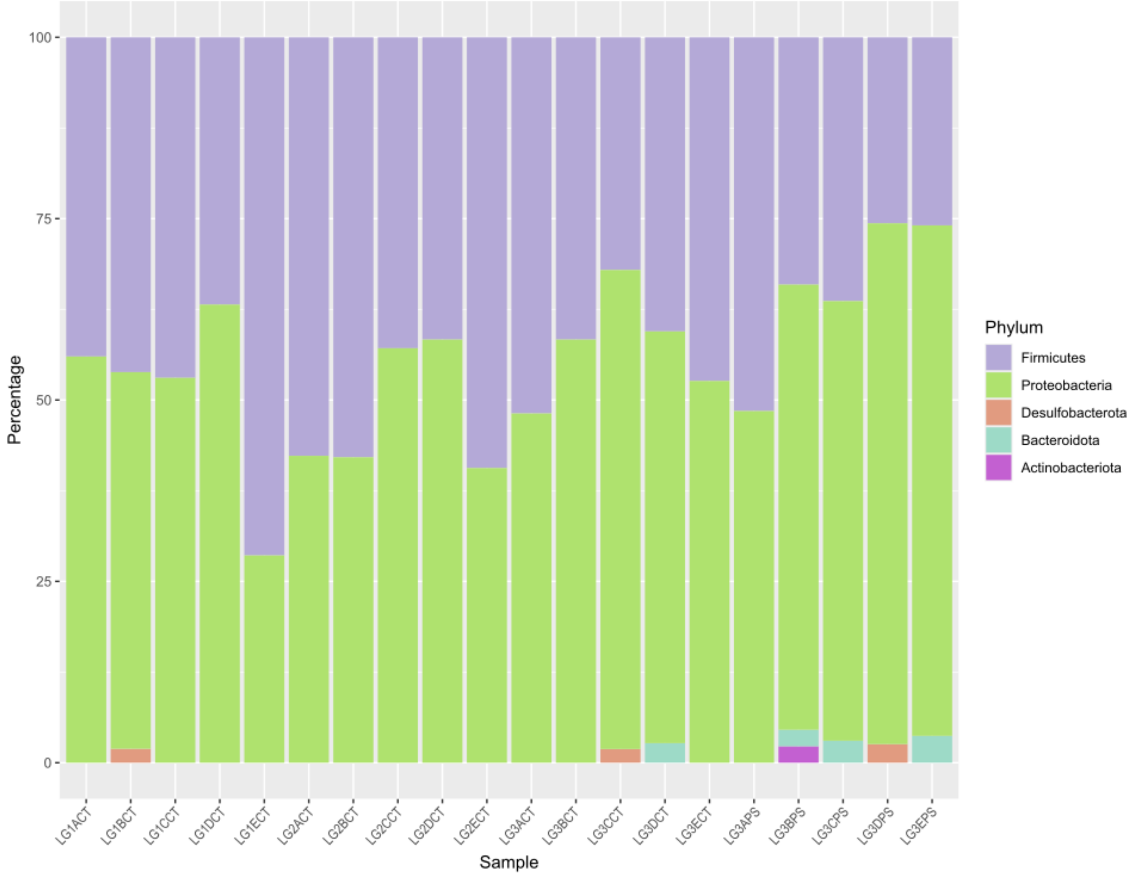

Supplementary Figure S6 - Phylum-level composition of the gut microbiota across larval instars. Relative distribution of bacterial phyla detected in larvae reared on control diet (CT) and polystyrene (PS) across the analyzed larval instars.

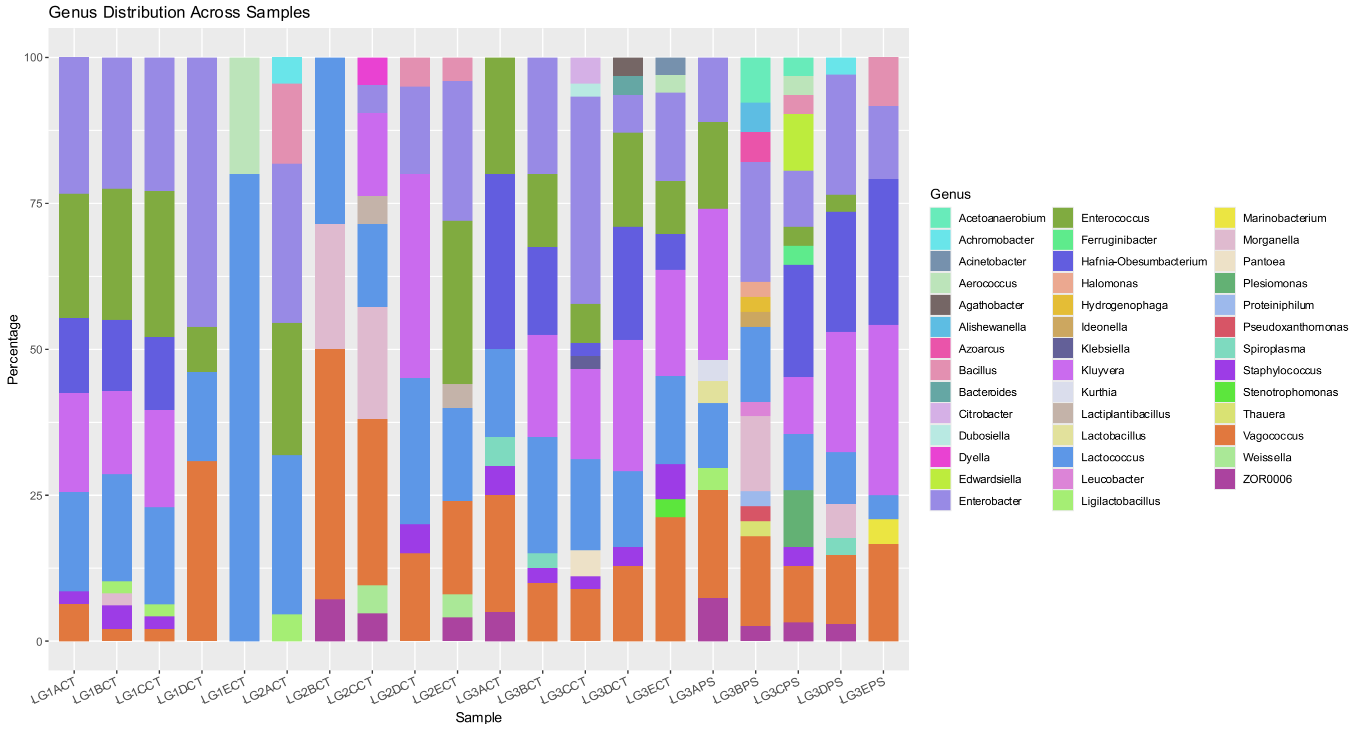

Supplementary Figure S7 – Genus level composition of the gut microbiota across larval instars. Taxa bar plot showing the distribution of bacterial genera across replicates and experimental groups in the first metabarcoding analysis. The analysis compared the gut microbiota of *A. diaperinus* larvae at different instars (LG1, LG2, LG3) reared on CT or PS diet.

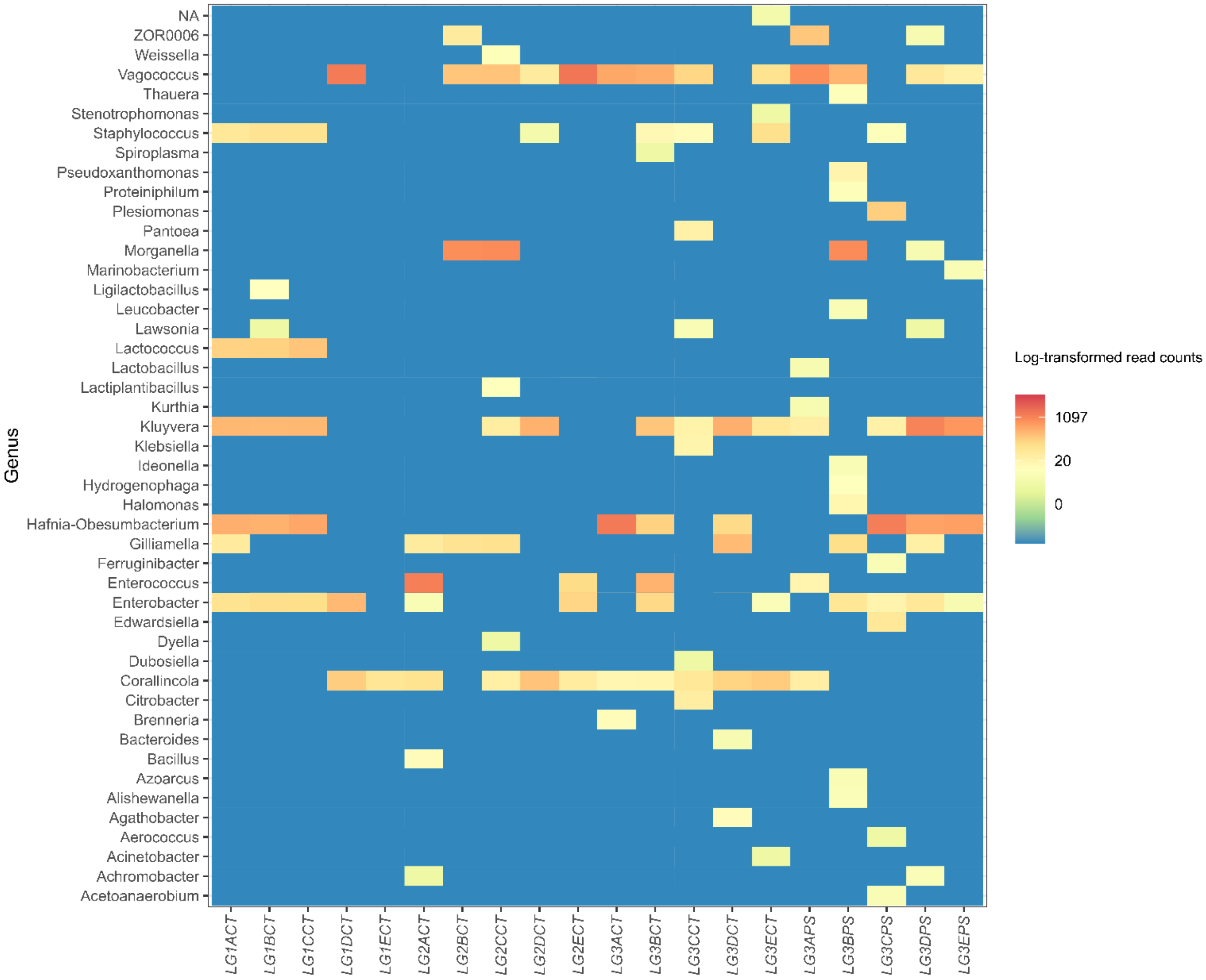

Supplementary Figure S8 - Genus abundance across larval instars.

Heatmap showing the relative abundance of bacterial genera in the metabarcoding analysis across larval instars. The x-axis represents 20 samples (five replicates per experimental group: LG1_CT, LG2_CT, LG3_CT, LG3_PS), and the y-axis lists the identified genera. The color gradient indicates log-scaled abundance levels.

A

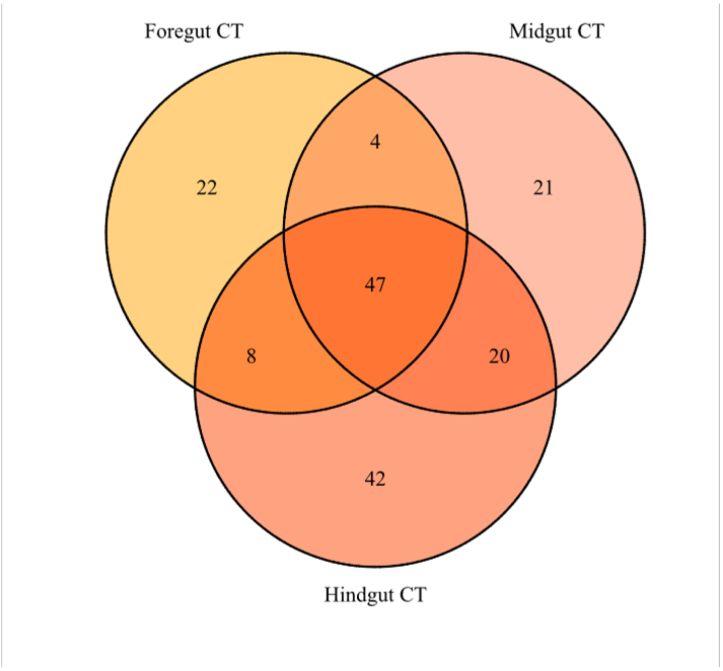

B

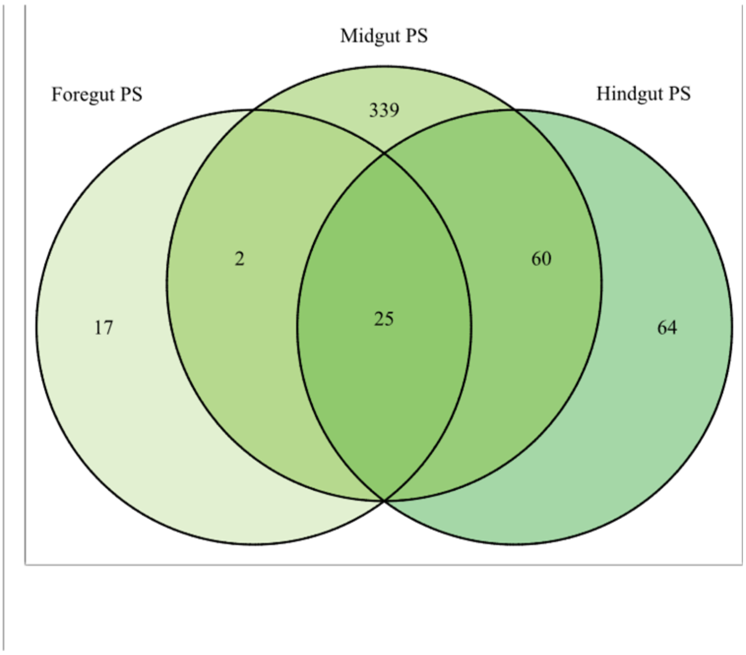

Supplementary Figure S9 – ASV distribution across gut sections.

Venn diagrams showing the distribution of ASVs in the second metabarcoding analysis. (A) Foregut, midgut, and hindgut of larvae reared on CT diet. (B) Foregut, midgut, and hindgut of larvae reared on the PS diet. Numbers indicate ASVs shared between or unique to each gut section.

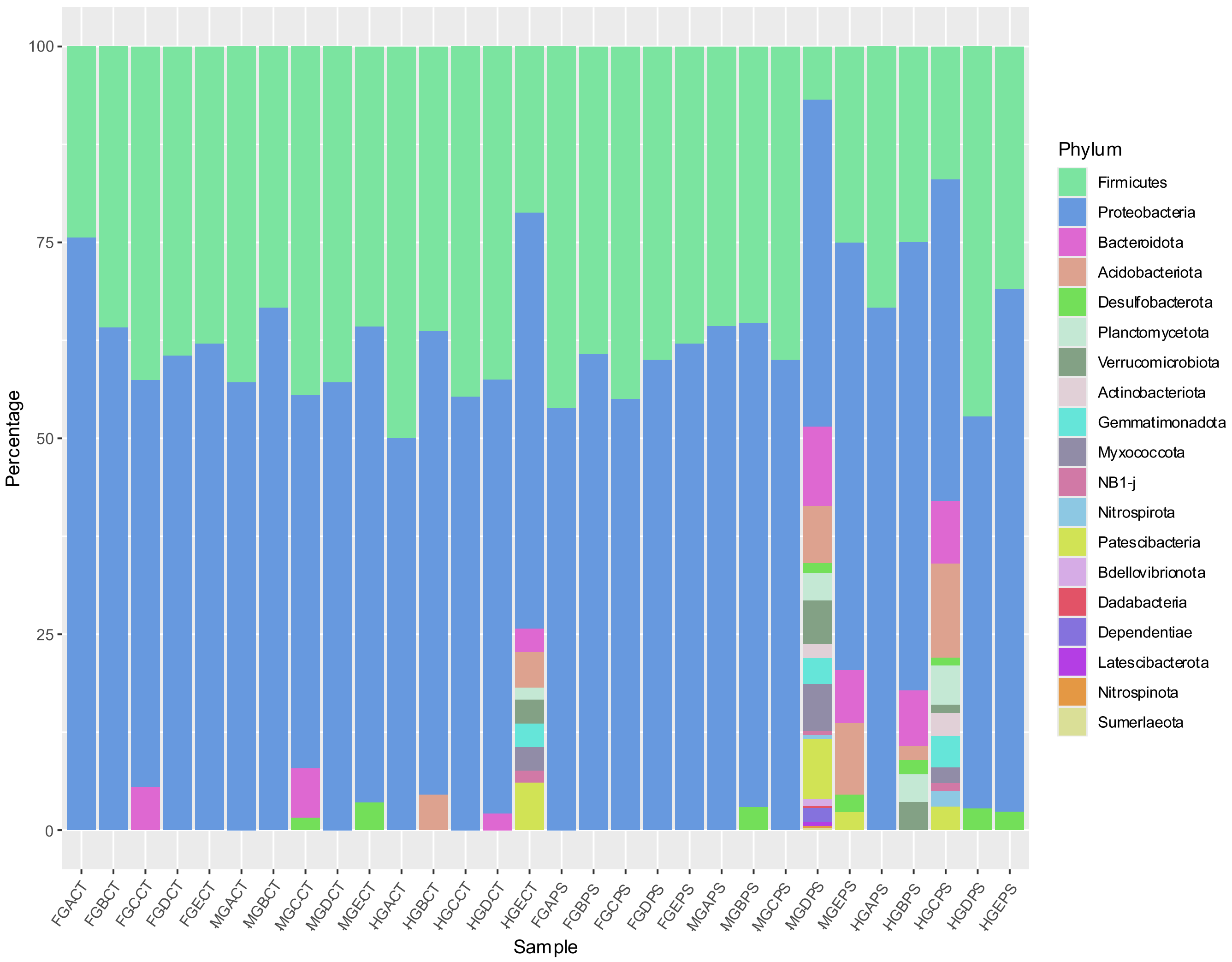

Supplementary Figure S10 - Phylum-level composition of the gut microbiota across gut sections. Relative distribution of bacterial phyla detected in foregut, midgut, and hindgut samples from larvae fed with control diet (CT) or polystyrene (PS).

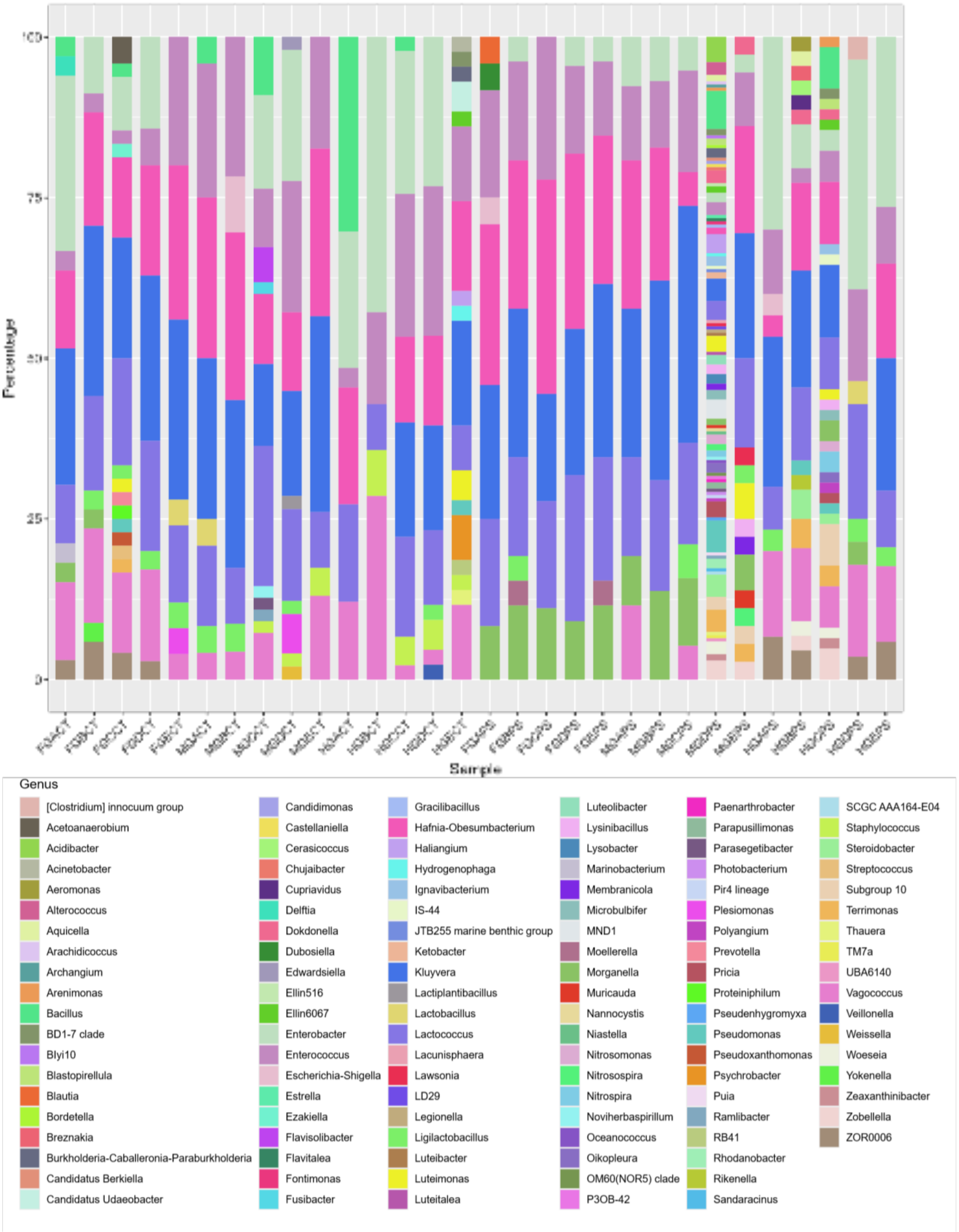

Supplementary Figure S11 – Genus level composition across gut sections.

Taxa bar plot showing the distribution of bacterial genera in the second metabarcoding analysis across gut sections (foregut, midgut, hindgut) of last instar *A. diaperinus* larvae reared on CT or PS diet.

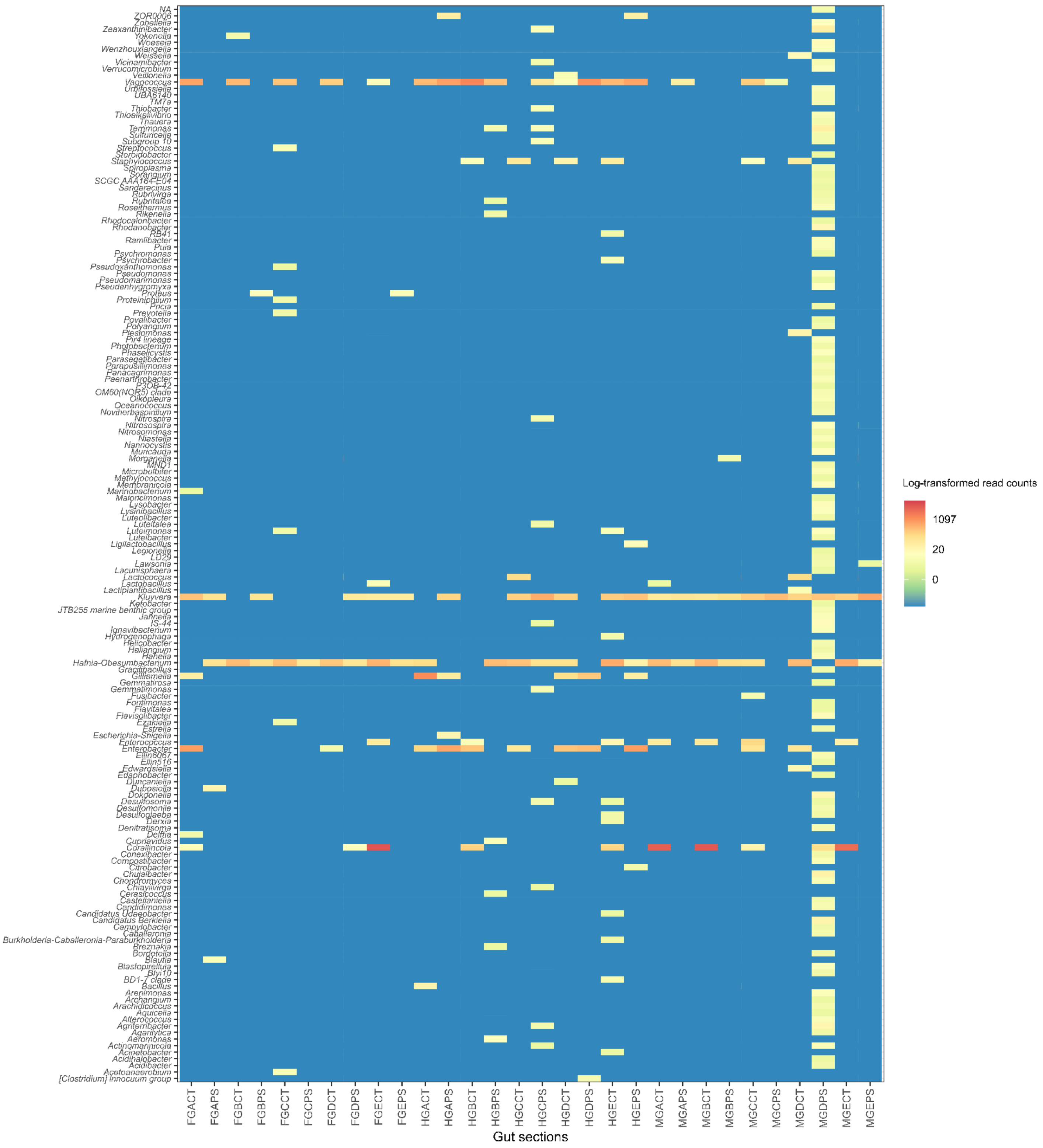

Supplementary Figure S12 - Heatmap of bacterial genera across gut sections.

Relative abundance of bacterial genera in the metabarcoding analysis of CT and PS fed larvae. The x-axis represents the 30 individual samples (five replicates per gut section and diet), while the y-axis lists the identified bacterial genera. The colour gradient (log-scaled) reflects the abundance levels of each genus.
